# Editorial: How close are we to understanding immune evasion and development of vaccines for mycobacterial pathogens?

**DOI:** 10.3389/fimmu.2026.1977361

**Published:** 2026-09-03

**Authors:** Jayne C. Hope, Natalia Elguezabal, William C. Davis

**Affiliations:** 1 The University of Edinburgh The Roslin Institute, Edinburgh, United Kingdom; 2 Animal Science Department, NEIKER Basque Institute for Agricultural Research and Development, Derio, Spain; 3 Washington State University, Pullman, Washington, United States

**Keywords:** disease, immunity, paratuberculosis, tuberculosis, vaccine

Mycobacterial diseases present a paradox for vaccinology: infection can generate strong and measurable immune responses, yet these responses frequently fail to eliminate the pathogen or prevent disease. The five articles in this Research Topic ‘*How close are we to understanding immune evasion and development of vaccines for mycobacterial pathogens?’* provide complementary perspectives on this challenge, spanning bovine tuberculosis (TB) and paratuberculosis examining mucosal immunology, vaccine delivery, host–pathogen interactions and the limitations of current models of protection. Collectively, they point towards a change in perspective: the critical question to address is not simply whether a vaccine induces an immune response, but whether it induces an *effective* immune response, in the appropriate place and at the optimal time.

A major theme across the topic is the ability of mycobacteria to persist in the face of substantial host immune activation. Prado-López et al., describe an example of this in naturally infected Holstein cattle with subclinical *Mycobacterium avium paratuberculosis* (MAP) infection. Whole-transcriptome analysis of peripheral blood identified a large number of differentially expressed genes, revealing robust innate immune activation alongside changes associated with RNA processing and translation inhibition. These findings suggest that MAP persistence involves a complex balance of host innate immunity activation and regulation, translational reprogramming, and metabolic adaptation. These novel insights illustrate the complexity of a host response that is simultaneously activated, but apparently unable to clear infection. Understanding this phenomenon of immune control without sterilizing protection may be critical for identifying biomarkers of disease progression and, ultimately, correlates of protective immunity.

The papers in the Research Topic highlight the importance of understanding immunity at the anatomical site of infection. Aguilar-Lopez et al., highlight the specialized and regionally heterogeneous nature of intestinal immunity during MAP infection. MAP crosses the intestinal barrier through Peyer’s patches and establishes infection within macrophages and dendritic cells, while simultaneously manipulating antigen presentation, inflammatory signaling and cellular function. A key point to consider is that systemic immune responses, including those of Th1 cells, do not necessarily translate into mucosal protection. Since the majority of studies of immune induction examine responses in the peripheral blood, fundamental questions for vaccine development remain: how does relevant-tissue immunity correlate with peripheral immune responses and are we measuring responses in the right place?

The two studies of Cuenca-Lara et al., and Tran et al., address vaccine-induced immunity to TB and provide evidence that the site of vaccination can substantially influence the quality of the immune response. In goats, Cuenca-Lara et al. demonstrated that intranasal Bacille Calmette-Guerin (BCG) vaccination can generate robust pro-inflammatory, antigen-specific responses both systemically and within the lung, highlighting the capacity to induce immunity at the primary site of infection. These responses include induction of antigen-specific T cells, pro-inflammatory cytokine expression as well as the activation and polarization of alveolar macrophages towards a pro-inflammatory phenotype. These findings support the concept that respiratory vaccination can establish immune activation at the portal of pathogen entry, enabling prevention of infection.

However, the study by Tran et al. provides an important counterpoint. A novel mucosal protein-only vaccine incorporating ESAT6 and CFP10 induced strong systemic and mucosal antibody responses, together with polyfunctional Th1 and Th17 responses and evidence of mycobacterial killing *in vitro*. However, this vaccine failed to significant reduction in pulmonary bacterial burden following challenge in mice, compared to BCG which induced significant protection. This demonstrates that strong immunogenicity is not necessarily sufficient to predict protective immunity *in vivo*. Antigenic breadth, the quality, duration and localization of immune responses, along with immune interactions within the infected tissue environment may all be critical.

Cardona's perspective on reinfection adds another layer of complexity. Experimental and epidemiological evidence suggests that repeated exposure to *M. tuberculosis* can alter both pathogen burden and host immunity. In a multiple-consecutive-infection model, repeated exposure increased pulmonary bacillary burden and boosted immune responses. Moreover, multiple exposures lessened the protective impact of BCG vaccination, compared to single pathogen challenge where BCG was efficacious. This may suggest that in endemic areas with high TB burden, BCG may provide lower protective benefit. This challenges conventional vaccine evaluation strategies based largely on a single exposure and highlights the importance of developing experimental models that better reproduce the complexity of natural transmission and exposure.

Taken together, these studies suggest that the next generation of mycobacterial vaccines will require more than new antigens or stronger adjuvants. We need a deeper understanding of protective immunity as a spatial and temporal phenomenon. For TB, this will require greater focus on generating durable lung-resident and mucosal responses prior to exposure. For MAP, it requires understanding how immunity is established and regulated across distinct intestinal compartments. Across both infections, there is a need to distinguish immune activation from effective immune control, and develop models that incorporate repeated exposure and the heterogeneous outcomes of natural infection.

A key message from the Research Topic is that the search for a single correlate of protection may be unrewarding. Protection is likely to emerge from a combination of cellular, humoral and innate responses, being active in the relevant tissue and at the critical point during infection. Integrating systems-level approaches, mucosal immunology, improved animal models and rational vaccine design offers a route towards identifying optimal strategies for induction of protective immunity.

The challenge for the field is therefore not simply to generate *more* immunity, but to generate *appropriately-targeted* immunity. Understanding how mycobacteria evade immune clearance—and how vaccination can place the host one step ahead at the site of infection—may ultimately be the key to transforming immunogenicity into durable protection.

## Dedication

This Research Topic, and editorial, is dedicated to the memory of Dr William (Bill) Davis, who sadly passed away on the 24^th^ July 2026. Bill was passionate about the study of mycobacterial pathogens, and worked tirelessly in studies of the immune response to both paratuberculosis and bovine TB, establishing himself as an international authority in the subject area. One of Bill’s greatest achievements was the foundation of the Washington State University Monoclonal Antibody Center, providing critical reagents to accelerate veterinary immunology research and vaccine development. In his honor, that facility has now been renamed the ‘William C. Davis Monoclonal Antibody Center’, ensuring his life-long contribution to veterinary immunology is permanently recognized. With his late wife Betty, Bill established an endowment supporting immunological research in food and companion animals, a commitment that will continue to benefit future generations of scientists. Bill’s dedication to veterinary immunology and his tireless research into mycobacterial diseases have left a lasting legacy. His scientific contributions were remarkable, but perhaps equally significant was his commitment to mentoring, inspiring collaboration and the many stimulating discussions and exchanges of ideas he fostered. He will be greatly missed by the veterinary immunology community, both as an exceptional scientist, and as a valued colleague and friend.

